# *Caliciopsismoriondi*, a new species for a fungus long confused with the pine pathogen *C.pinea*

**DOI:** 10.3897/mycokeys.73.53028

**Published:** 2020-09-25

**Authors:** Duccio Migliorini, Nicola Luchi, Alessia Lucia Pepori, Francesco Pecori, Chiara Aglietti, Fabio Maccioni, Isabel Munck, Stephen Wyka, Kirk Broders, Michael J. Wingfield, Alberto Santini

**Affiliations:** 1 Institute for Sustainable Plant Protection – National Research Council (IPSP-CNR), Via Madonna del Piano 10, I-50019, Sesto Fiorentino, Firenze, Italy; 2 Department of Agrifood Production and Environmental Sciences, University of Florence, Piazzale delle Cascine 28, I-50144, Firenze, Italy; 3 Forest Health Protection, USDA Forest Service, 271 Mast Road, Durham, NH 03824, Durham, USA; 4 Department of Bioagricultural Sciences and Pest Management, Colorado State University, Fort Collins, Colorado, USA; 5 Smithsonian Tropical Research Institute, Apartado 0843-03092, Balboa, Ancon, Panama; 6 Forestry and Agricultural Biotechnology Institute (FABI), University of Pretoria, Private Bag X20, Pretoria 0028, South Africa

**Keywords:** *Caliciopsis* canker, *Caliciopsis* spp., forest pathogen, one new species, pine trees, taxonomy

## Abstract

The genus *Caliciopsis* (Eurotiomycetes, Coryneliales) includes saprobic and plant pathogenic species. Caliciopsis canker is caused by *Caliciopsispinea* Peck, a species first reported in the 19^th^ century in North America. In recent years, increasing numbers of outbreaks of Caliciopsis canker have been reported on different *Pinus* spp. in the eastern USA. In Europe, the disease has only occasionally been reported causing cankers, mostly on *Pinusradiata* in stressed plantations. The aim of this study was to clarify the taxonomy of *Caliciopsis* specimens collected from infected *Pinus* spp. in Europe and North America using an integrative approach, combining morphology and phylogenetic analyses of three loci. The pathogenicity of the fungus was also considered. Two distinct groups were evident, based on morphology and multilocus phylogenetic analyses. These represent the known pathogen *Caliciopsispinea* that occurs in North America and a morphologically similar, but phylogenetically distinct, species described here as *Caliciopsismoriondi***sp. nov.**, found in Europe and at least one location in eastern North America. *Caliciopsismoriondi* differs from *C.pinea* in various morphological features including the length of the ascomata, as well as their distribution on the stromata.

## Introduction

Species in the Coryneliaceae (Eurotiomycetes) have a worldwide distribution; they occur in both hemispheres and in both temperate and tropical climates (Fitzpatrick 1942). The family accommodates seven genera including *Caliciopsis* Peck, *Corynelia* Ach. ex Fr., *Coryneliopsis* Butin, *Coryneliospora* Fitzp., *Fitzpatrickella* Benny, Samuelson & Kimbr., *Hypsotheca* Ellis & Everh., *Lagenulopsis* Fitzp. and *Tripospora* Sacc. (Crous et al. 2019; Wood et al. 2016; Wijayawardene et al. 2020). The genus *Caliciopsis* includes both saprobic and plant pathogenic species. Recently, a taxonomic key for the genus *Caliciopsis* has been presented (Garrido-Benavent and Perez-Ortega 2015). *Caliciopsisnigra* [now recombined to *Hypsothecanigra* (Crous et al. 2019)] is the causal agent of cankers on stems of the Mediterranean cypress, *Cupressussempervirens* and common juniper, *Juniperuscommunis* (Intini 1980) and *C.indica* is a pathogen on *Garciniaindica* leaves (Pratibha et al. 2010). *Caliciopsisarceuthobii* infects the flowers of several species of dwarf mistletoe in the genus *Arceuthobium* (Ramsfield et al. 2009), *Caliciopsisrhoina* is associated with bark and trunk cankers on *Toonasinensis* (Rikkinen 2000), while *Caliciopsisbrevipes* was reported on needles and bark of *Araucariaaraucana* and *C.cochlearia* on needles and twigs of *A.araucana*, *Fitzroyacupressoides*, *Austrocedruschilensis*, *Pilgerodendronuviferum* and *Podocarpusnubigenus* (Butin 1970). More recently, *Caliciopsispleomorpha* [now recombined in *Hypsotheca* as *H.pleomorpha* (Crous et al. 2019)] has been reported as the causal agent of a canker disease on various *Eucalyptus* species in Australia (Pascoe et al. 2018).

Caliciopsis canker has been reported as an emerging disease of *Pinus* in the eastern USA (Munck et al. 2015, 2016; Costanza et al. 2018; Haines et al. 2018; Schulz et al. 2018a, b; Whitney et al. 2018) and is caused by the fungus *Caliciopsispinea*. The pathogen gives rise to cankers and abundant resin bleeding on branches and stems of young and mature *Pinusstrobus* trees, which can lead to crown wilting and defoliation and, in some cases, killing significant portions of the tree crowns. In the USA, *C.pinea* has been known since at least 1920 (Fitzpatrick 1920). It was considered “not uncommon” in eastern North America on *P.strobus* and on various conifer species in western North America (Ray 1936). After the accidental introduction of white pine blister rust (*Cronartiumribicola* J.C. Fisch.) into the USA in the early 1900s, Caliciopsis canker was ignored.

*Caliciopsispinea* is considered native to North America (Harrison 2009). In Europe, Lanier (1965) reported this species for the first time from France on *P.halepensis*, *P.insignis*, *P.nigra* and *P.pinaster*, although the pathogen had been known in the region since the late 1800s (Rehm 1896). The disease was also reported in Italy (Capretti 1978, 1980) on several different *Pinus* species, both native and non-native. While all of these reports of Caliciopsis canker have been attributed to *C.pinea*, a recent study by Munck et al. (2015) suggested that a closely related, but distinct lineage of the fungus was present on *P.nigra* and *P.radiata* in Italy. Recently, an extensive survey of pine plantations in central-south Italy revealed several *P.radiata* stands showing crown yellowing, die-back and profuse resin production on the stems and shoots associated with depressed cankers. Fungal fruiting bodies resembling those of *Caliciopsis* in all stages of development were found on the cankered tissues (N. Luchi unpublished data).

No comprensive phylogenetic study has been undertaken on *Caliciopsis* spp. associated with cankers on *Pinus* spp. including both Europe and North America. Given the findings of Munck et al. (2015), it is possible that a distinct species of *Caliciopsis* is present in Europe. Furthermore, this fungus could have a host range, ecology and epidemiology different to those of its North American congener. The aim of this study was to compare pine-infecting *Caliciopsis* isolates from Europe and North America, based on morphological features and phylogenetic inference and to determine whether these represent a single or more than one species.

## Materials and methods

### Sampling and isolation of fungal strains

Isolates used in this study were obtained from a number of surveys of Caliciopsis canker on native and non-native *Pinus* spp. in plantations and naturally regenerated eastern white pine stands growing in different areas of Europe (EU) and North America (NA). Isolates from NA were obtained from Georgia, North Carolina, Tennessee and Virginia (this study) and Maine, Massachusetts, New Hampshire in the USA (Munck et al. 2015). Isolates from EU were obtained from France, Italy and Spain (Table 1).

**Table 1.** List of *Caliciopsis* spp. and *Corynelia* spp. used in comparisons of the morphology and culture characteristics and phylogenetic analyses and inoculation tests in this study.

**Species**	**Isolate ID**	**Substrate**	**Location^1^**	**GenBank accession numbers**		
				**ITS**	**EF1-α**	**Bt1**
*Caliciopsispinea*	US 27	*Pinusstrobus*	Blackwater, NH, USA	KY099598	MK913567	MN150097
	US 42	*P.strobus*	Farmington, NH. USA	MK785367	MK913566	MN150096
	US 52	*P.strobus*	Bethel, ME, USA	MK785366	MK913565	MN150095
	US 67	*P.strobus*	Greenfield, NH, USA	MK785365	MK913564	MN150098
	US 71	*P.strobus*	Parsonsfield, ME, USA	MK785364	MK913563	MN150101
	US 76	*P.strobus*	Bear Brook, NH, USA	MK785363	MK913562	MN150102
	US 81	*P.strobus*	West Groton, MA, USA	KY099601	MK913561	MN150094
	US 100	*P.strobus*	Merrimack, NH, USA	MK785361	MK913560	MN150092
	US 110	*P.strobus*	Burns Farm, Milford, NH, USA	MK785360	MK913559	MN150091
	US 124	*P.strobus*	Albany, ME, USA	MK785359	MK913558	MN150090
	US 137	*P.strobus*	Alternate Brownfield, ME, USA	MK785358	MK913557	MN150089
	US 139	*P.strobus*	Sebago Lake, ME, USA	MK785357	MK913556	MN150100
	US 149	*P.strobus*	Brownfield, ME, USA	MK785356	MK913555	MN150088
	US 151	*P.strobus*	Little Ossipee Farm, Livington, USA	MK785355	MK913554	MN150087
	US 161	*P.strobus*	Androscoggin River Park, ME, USA	MK785354	MK913553	MN150086
	US 163	*P.strobus*	Androscoggin River Park, ME, USA	MK785353	MK913552	MN150085
	US 167	*P.strobus*	Bowdoinham, ME, USA	MK785352	MK913551	MN150084
	US 172	*P.strobus*	Naples, ME, USA	MK785351	MK913550	MN150083
	US 199	*P.strobus*	Sauford, ME, USA	MK785350	MK913549	MN150082
	US 206	*P.strobus*	Androscoggin River Park, ME, USA	MK785349	MK913548	MN150081
	US 220	*P.strobus*	New Castle, ME, USA	MK785348	MK913547	MN150080
	US 222b	*P.strobus*	Palmer, MA, USA	MK785347	MK913546	MN150099
	US 225a	*P.strobus*	Douglas, MA, USA	MK785346	MK913545	MN150078
	US 230d	*P.strobus*	Peru, ME, USA	KY099602	MK913544	MN150077
	US 232b	*P.strobus*	Barre, MA, USA	MK785344	MK913543	MN150076
	US 234a	*P.strobus*	Hollis, NH, USA	MK785343	MK913542	MN150075
	US 237	*P.strobus*	Macon, NC, USA	MK785342	MK913541	MN150074
	US 238	*P.strobus*	Neola, VA, USA	MK785341	MK913540	MN150073
	US 240	*P.strobus*	Lyme, NH, USA	MK785340	MK913539	MN150072
	US 252	*P.strobus*	USA	MK785339	MK913538	MN150071
	US 255	*P.strobus*	Unicio State Park, GA, USA	MK785338	MK913537	MN150070
	US 256	*P.strobus*	Wartburg, TN, USA	MK785336	MK913536	MN150069
	US 257	*P.strobus*	Unicio State Park, GA, USA	MK785336	MK913535	MN150079
*C.moriondi*	IT 1, CBS 146717	*P.radiata*	Carcheri, Tuscany, Italy	MN156540	MK913586	MN150120
	IT 2	*P.radiata*	Carcheri, Tuscany, Italy	MK785385	MK913585	MN150119
	IT 4	*P.radiata*	Carcheri, Tuscany, Italy	MK785384	MK913584	MN150118
	IT 5	*P.radiata*	Carcheri, Tuscany, Italy	MK785383	MK913583	MN150117
	IT 6	*P.radiata*	Carcheri, Tuscany, Italy	MK785382	MK913582	MN150116
	IT 7	*P.radiata*	Carcheri, Tuscany, Italy	MK785381	MK913581	MN150115
	IT 9	*P.radiata*	Carcheri, Tuscany, Italy	MK785380	MK913580	MN150114
	IT 11	*P.radiata*	Carcheri, Tuscany, Italy	MK785379	MK913579	MN150113
	IT 13	*P.radiata*	Carcheri, Tuscany, Italy	MK785378	MK913578	MN150112
	IT 14	*P.radiata*	Carcheri, Tuscany, Italy	MK785377	MK913577	MN150111
	IT 15	*P.radiata*	Carcheri, Tuscany, Italy	MK785376	MK913576	MN150110
	SP 1	*P.radiata*	San Sebastian de Garabandal, Spain	MK785372	MK913571	MN150106
	IT 17	*P.nigra*	Antella,Tuscany, Italy	MK785375	MK913575	MN150109
	IT 20	*P.radiata*	Carcheri, Tuscany, Italy	MK785374	MK913574	MN150108
	IT 22	*P.radiata*	Fucecchio, Tuscany, Italy	MK785373	MK913573	MN150107
	LSVN1233	*P.radiata*	Pyrénées Atlantiques, France	MK785386	MK913572	MN150121
	US 64	*P.resinosa*	Bear Brook State Park, NH, USA	MK785371	MK913570	MN150105
	US 65	*P.resinosa*	Bear Brook State Park, NH, USA	MK785370	MK913569	MN150104
	US 66	*P.resinosa*	Bear Brook State Park, NH, USA	MK785369	MK913568	MN150103
*C.orientalis*	CBS 138.64	*Tsugacanadiensis*	Nashville, Canada	KP881690	MK91358	MN150122
*C.pinea*	CBS 139.64	*P.strobus*	Chalk River, Canada	KP881691	DQ677937	MN150093
*C.pseudotsugae*	CBS 140.64	*P.menziesii*	Cowichan Lake, Canada	MK785387	MK913587	MN150123
*C.beckhausii* ^*^	MA 18186	Quercusilexsubsp.rotundifolia	Spain	NR132090		
*C.calicioides* ^*^	211	*Populustrichocarpa*	Wentachee National Forest, WA, USA	JX968549		
*C.eucalypti* ^*^		*Eucalyptusmarginata*	Western Australia, Australia	KY173391		
*C.indica* ^*^	GFCC 4947	*Garciniaindica*	India	NR119752		
C.valentina ^*^		Quercusilexsubsp.rotundifolia	Spain	NR132091		
*Coryneliauberata* ^*^	ARW 686	*Afrocarpusfalcatus*	Western Cape, South Africa	KP881707		
*Co.fructigena* ^*^	ARW 250	*Podocarpuslatifolius*	Western Cape, South Africa	KP881704		
*Co.africana* ^*^	ARW 247	*Podocarpuslatifolius*	Western Cape, South Africa	KP881693		
*Hypsothecapleomorpha* ^*^	VPRI 15646	*Eucalyptus*	Australia	MG641785		
*Lagenulopsisbispora* ^*^	ARW 249	*Podocarpuslatifolius*	Western Cape, South Africa	KP881709		

^*^ ITS sequences obtain from GenBank. ^1^ Canada (CA), France (FR), Italy (IT), Spain (SP) and United States of America (USA). States abbreviations are Georgia (GA), Maine (ME), Massachusetts (MA), New Hampshire (NH), North Carolina (NC), Tennessee (TE), Virginia (VA), and Washington (WA).

Samples from Italy were obtained from pine trees with Caliciopsis canker symptoms from three different locations in Tuscany (Central Italy). Five shoots with Caliciopsis canker from five different trees were collected at each of the three Italian sites. Other isolates from *Pinus* and other host species used in this study included *Caliciopsispinea* LSVN1233 (from France, supplied by Dr. R. Ioos), *C.pinea*SP 1 (from Spain, supplied by Dr. P. Capretti), *C.pinea* CBS 139.64 (from Canada), *C.orientalis* CBS 138.64 (from Canada) and *C.pseudotsugae* CBS 140.64 (from Canada). All isolates are maintained in the culture collections of the Institute for Sustainable Plant Protection – National Research Council (IPSP-CNR, Italy) and the Department of Bioagricultural Sciences, Colorado State University.

Samples were placed in paper bags and transferred to the laboratory for isolation. Pine twigs (5 cm long; 0.5 to 1 cm diameter) were surface disinfested with 75% ethanol (1 min) and 3% sodium hypochorite (NaOCl) (3 min), after which they were rinsed three times in sterile water. A sterile scalpel was used to remove the outer portions. Necrotic tissues were cut in small pieces and placed in 90-mm Petri dishes containing 1.5% Potato Dextrose Agar (PDA, DIFCO, Detroit, Michigan, USA), amended with streptomycin (0.050 g/l). All plates were incubated in the dark at 20 °C for 10–15 days. Fungal colonies with a morphology resembling *C.pinea* were transferred to fresh plates to obtain pure cultures.

### Morphology and culture characteristics

Caliciopsis fruiting bodies on cankered bark of the Italian specimens were mounted on glass slides in 80% lactic acid, amended with bromothymol blue and examined using a dissection microscope (SMZ800, Nikon, Japan). The length and width of 50 released ascospores and 30 fruiting bodies were measured under a light microscope (Axioskop 50 Zeiss, Germany) and images captured with a Nikon Digital Sight DS-5M camera (Nikon Instruments Software-Elements Basic Research). The means and range dimensions of fruiting bodies and ascospores were compared with those reported in literature (Table 2). The morphology of fungal colonies was determined for cultures grown for four weeks on 1.5% Malt Extract Agar (MEA, DIFCO, Detroit, Michigan, USA) and 1.5% PDA.

**Table 2.** Morphological characteristics of *C.pinea* isolates described in literature and in this study and compared with *C.moriondi*. Measurements are presented as height × width, both measured reported as (min value) mean+/-SD (max value).

	*C.moriondi*	*C.pinea*	*C.pinea*	*C.pinea*	*C.pinea*	*C.pinea*	*C.pinea*
Host	*P.radiata*	*P.radiata*	*P.pinaster*	P.nigravar.austriaca	*P.mugo*	*P.strobus*	*P.pinaster*
Reference	This study	Capretti 1978	Capretti 1978	Capretti 1980	Rehm 1896	Fitzpatrick 1920	Lanier 1965
Sampling location	France, Italy, Spain, USA (New Hampshire)	Italy	Italy	Italy	Germany	Eastern North America	France
Ascomata height	(450) 845±24 (1240) µm	2–5mm	2–5mm	–		1–3 mm	2–3 mm
Stalk width	(51) 79±2 (135) µm	–	–	–	100–140 µm	100–125 µm	–
Ascigerous swelling	terminal	Apical	Apical	Apical	Apical	Apical	Apical or sub-apical
Swelling size	(106)281±8(406) × (81)142±5(268) µm	–	–	–	400 × 175–275 µm	175 µm in diameter	–
Ascospore shape	Small, oval	Small, oval	Small, oval	Small, oval	Ellipsoidal to ovoidal or globose	Ellipsoidal	Ellipsoidal, brown-hyaline
Ascospore size	(3)4.4±0.07(6.2) × (1.8)2.5±0.04(3.5) µm	(3.5)4.4(6.3) × (3.1)3.4(4) µm	(3.5)4.6(6.3) × (3.1)3.7(4.2) µm	(3.7)5.3(7.9)× (2.8)4.2(4.4) µm	3.5–6 × 2–4 µm	5–6 × 3 µm	5–6 × 3–3.5 µm
Asci size	(26)37±6(53) × (5.3)6.3±0.4(7.4) µm	–	–	–	12–17 × 5–8 µm	20 × 8 µm	–
Colonies on malt agar	White-hyaline appressed to the agar. Turning to brown in time.	White-brown with frequent anastomoses	White-brown with frequent anastomoses	–	–	* 1	–

### Daily growth rate in culture

Six-mm diameter mycelial plugs were taken from the margins of actively growing seven-day-old colonies on PDA, using a flame-sterilised cork borer and were placed at the centres of 90-mm Petri dishes containing 1.5% PDA or 1.5% MEA. Five replicates were used for each of five selected strains (IT6, IT9, IT16, IT17, IT22) at each temperature. Dishes were incubated at 4 °C, 15 °C and 20 °C. Two measurements of colony diameter perpendicular to each other were made at 3, 7, 14, 21 and 28 days and daily growth rate was calculated as an average for each strain on each substrate. Data were analysed using a factorial ANOVA, considering temperature and substrate as the independent factor and daily growth rate as the dependent factor.

### DNA extraction and PCR amplification

Fungal isolates, including those from the Westerdijk Fungal Biodiversity Institue (CBS 138.64, CBS 139.64 and CBS 140.64) listed in Table 1, were grown in 90-mm Petri dishes containing MEA covered with 300PT cellophane membrane (Celsa, Varese, Italy) and incubated at 20 °C in the dark for 10 days. Fresh mycelium (ca. 80 mg) was scraped from the surface of the cellophane and ground in 2 ml microfuge tubes with two tungsten beads (3 mm) (Qiagen, Hilden, Germany) and 400 µl Buffer P1 (EZNA Plant DNA Kit, Omega Bio-tek, Norcross, GA, USA) using a Mixer Mill 300 (Qiagen, Hilden, Germany) [2 min; 20 Hz]. DNA was extracted from all samples using the EZNA Plant DNA Kit (Omega Bio-tek, Norcross, Georgia, USA), following the manufacturer’s instructions. The DNA concentration was measured using a Nanodrop ND-1000 spectrophotometer (NanoDrop Technologies, Wilmington, Delaware, USA).

For the phylogenetic analyses, partial regions of three loci were amplified. Amplification of the internal transcribed spacer ITS region (including spacers ITS1 and ITS2 and the 5.8S gene of the rDNA) was done using primers ITS1 and ITS4 (White et al. 1990), of β-tubulin 1 (Bt1) gene using primers Bt2a and Bt2b (Glass and Donaldson 1995) and of translation elongation factor 1-α (EF1-α) gene using primers EF1-983F and EF-gr (Rehner and Buckley 2005). PCR reaction mixtures (25 µl) contained 1 µl of genomic DNA, 2.5 µl of each 10 µM primer, 0.5 µl of 10 mM dNTPs (GeneSpin, Milan, Italy), 2.5 µl of 10X PCR Buffer (GeneSpin), 3 µl of 25 nM MgCl and 0.5 µl (5 U/µl) of Taq polymerase (TaqPol, GeneSpin). Ampliﬁcations were carried out in a Mastercycler (Eppendorf, Hamburg, Germany) using the following PCR conditions: for ITS and Bt1: initial denaturation at 95 °C for 5 min; followed by 35 cycles of denaturation at 94 °C for 90 sec, annealing at 56 °C for 1 min and extention at 72 °C for 2 min; and a final elongation step at 72 °C for 10 min. For EF1-α: initial denaturation at 95 °C for 8 min; followed by 35 cycles of denaturation at 95 °C for 30 sec, annealing at 55 °C for 30 sec and extention at 72 °C for 1 min; and a final elongation step at 72 °C for 5 min (modified from Pepori et al. 2015). Ampliﬁcation products were separated by electrophoresis on gels containing 1% (w/v) of agarose LE (GeneSpin). The approximate length (bp) of the ampliﬁcation products was determined using the 100-bp DNA ladder Ready to Load (Genespin). PCR amplicons were puriﬁed with a miPCR Puriﬁcation Kit (Metabion International, Planegg, Germany) and sequenced in both directions at Macrogen (Seoul, South Korea). The quality of amplified nucleotide sequences was checked with the software package Geneious version 10.0.9 (Biomatters, Auckland, New Zealand). Generated sequences were submitted to NCBI GenBank (Table 1).

### Multi-locus phylogenetic analyses

BLAST searches of the generated sequences were done against the NCBI GenBank database (https://blast.ncbi.nlm.nih.gov/Blast.cgi) to identify the most closely-related sequences. Sequences were compared to those of known *Caliciopsis* species and other Coryneliaceae obtained from GenBank: ITS sequences of *Caliciopsisbeckhausii* (NR_132090), *C.calicioides* (JX968549), *C.eucalypti* (KY173391), *C.indica* (NR_119752), *C.orientalis* (KP881690), *C.pinea* (KP881691, KY099598, KY099601, KY099602), *Hypsotecapleomorpha* (MG641785), *C.valentina* (NR_132091), *Coryneliauberata* (KP881707), *Co.fructigena* (KP881704), *Co.africana* (KP881693), *Lagenulopsisbispora* (KP881709); EF1-α sequence of *Caliciopsispinea* (DQ677937). *Lagenulopsisbispora* (KP881709) was selected as outgroup in the ITS dataset, whereas *C.orientalis* (CBS 138.64) and *C.pseudotsugae* (CBS 140.64) were selected as outgroup taxa in the EF1-α and Bt1 datasets. The software package Geneious (Auckland, New Zealand) was used for manual optimisation and alignment (ClustalW) of the sequences. Gaps were treated as missing data.

Phylogenetic analyses of all obtained sequences were performed using MEGA 7 (Kumar et al. 2016), Maximum Parsimony (MP) was performed using the heuristic research option with random stepwise addition with 1000 replicates, tree bisection and reconnection (TBR) as branch swapping algorithm and random taxon addition of sequences for the construction of MP trees. All characters were weighted equally and character state transitions were treated as unordered. Parameters measured for parsimony included tree length (TL), consistency index (CI), rescaled consistency index (RC), retention index (RI) and homoplasy index (HI). Bootstrap support values were evaluated using 5000 bootstrap replicates (Hills and Bull 1993).

Datasets were also analysed by Bayesian Inference (BI) using MrBayes 3.1.2 (Ronquist and Huelsenbeck 2003), with a General Time Reversible (GTR) model and gamma distributed rate variation across sites. Six Markov Chain Monte Carlo (MCMC) chains (Larget and Simon 1999) were run for 3 million generations, starting from a random tree and using the default temperature. Every 100^th^ tree was sampled and the first 100,000 generations were discarded as burn-in. The burn-in period was determined after testing for stationarity of likelihood values, that is, by plotting numbers of generation versus the log probability and checking for the convergent diagnostic PSRF, which approached 1 (Ronquist et al. 2005). The consensus tree was calculated in MrBayes with the command sumt (Ronquist et al. 2005). The resulting phylogenetic tree was visualised using TreeView (Page 1996) and edited in TreeGraph2 (Stöver and Müller 2010).

### Inoculation tests

An inoculation experiment was carried out at the IPSP-CNR nursery facilities, located at Antella, Province of Florence, Italy (43°43'N, 11°22'E; 170 m a.s.l.). Three-year-old seedlings of *Pinushalepensis*, *P.pinaster* and *P.pinea*, with 36 plants per species, were planted in a randomised block design. The plants were maintained in rows 1 m apart and grown in a substrate comprised of commercially-produced loam and drip irrigated. The site had been completely cleared and ploughed prior to planting and was weeded each month.

Inoculations were performed in June 2014. A 6-mm diameter cork borer was used to remove the bark and expose the cambium on each plant. A plug of mycelium of the test fungus that had been grown in Petri dishes on 1.5% PDA for 20 days at 25 °C in the dark was inserted, with the mycelium side placed downwards into each wound. For inoculations, four different Italian *Caliciopsis* isolates (IT5, IT7, IT20 and IT22), recovered from infected *Pinus* sp. in the field, were used (Table 1). The inoculation trial was performed using eight trees per isolate on each of the *Pinus* spp. Four plants for each *Pinus* sp. were mock-inoculated using sterile PDA as controls.

Pathogenicity was assessed, based on the length of lesions (mm) after six months. Statistical analyses were performed by using Statistica 10.0 (StatSoft Inc. 1984–2011). To fulfil Kock’s postulates, re-isolations were carried out from the lesions on all the inoculated and control plants.

## Results

### Morphology and culture characteristics

**Figure 1.**
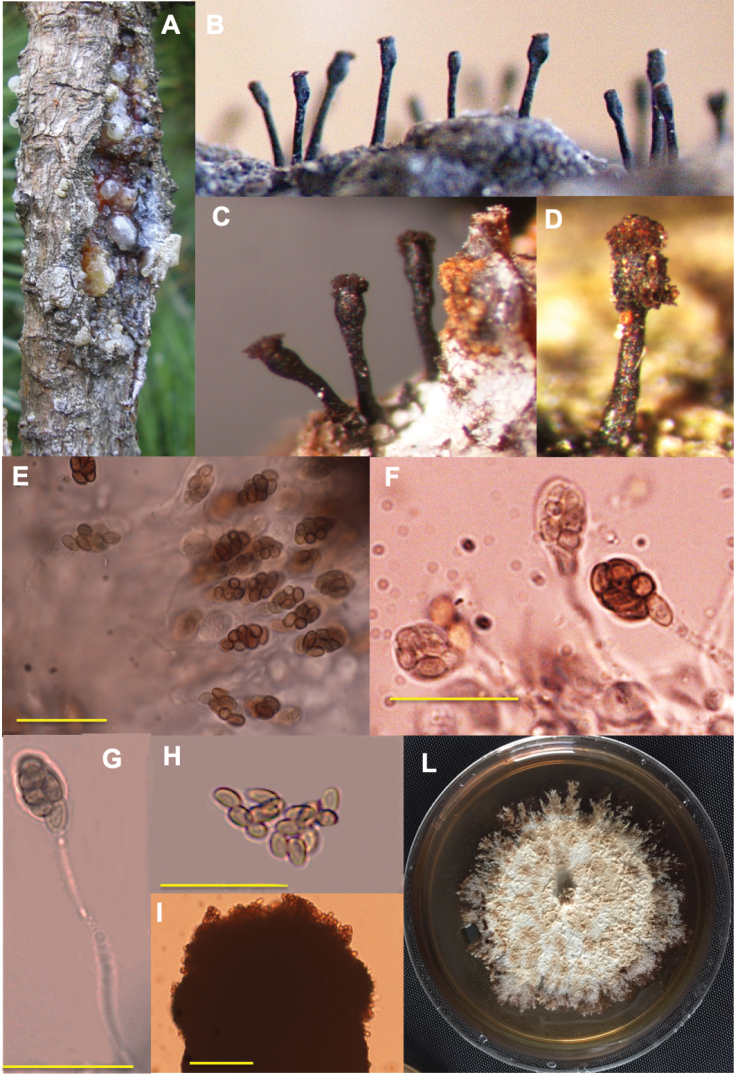
*Caliciopsismoriondi* structures **A** cankers on a *Pinusradiata* trunk **B–D** ascomata growing from a canker **B** image of *C.pinea* ascigerous columns from an archive of 1970 (provided by Prof. Paolo Capretti) **E–G** asci **H** ascospores **I** ascigerous, terminal portion **L** four weeks colony grown at 20 °C on MEA. Scale bars: 2.5 µm (**F–H**), 5 µm (**I**).

**Figure 2.**
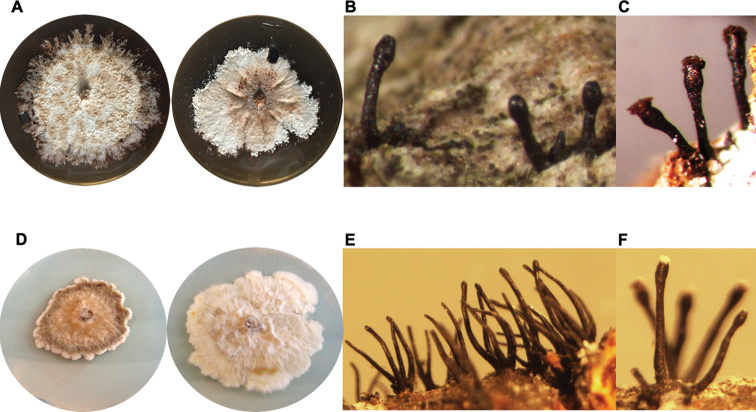
Morphological differences between *Caliciopsismoriondi* and *C.pinea***A–C***Caliciopsismoriondi*: **A** four-week-old colonies grown at 20 °C on MEA **B, C** ascomata growing from a canker of *Pinusradiata***D–F***Caliciopsispinea*: **D** four-week-old colonies grown at 20 °C on MEA **E, F** ascomata growing from a canker of *P.radiata*.

Fruiting bodies on bark taken from infected trees were black ascomata assembled in tufts with ascigerous swellings at the apices containing ascospores. The Italian specimens had different morphological characteristics from those reported in literature for *Caliciopsispinea* (Table 2, Figures 1, 2). Colonies of the Italian strains grown on MEA were white, appressed to the agar surface, turning to brown with time.

### Growth in culture

No growth was detected for any isolate at 4 °C. Isolates showed significantly greater growth at 20 °C. Mean daily growth rate (mm/day) at 4 °C = 0 ± 0; at 15 °C = 0.037 ± 0.017; at 20 °C = 0.076 ± 0.028; F = 412.371; p < 0.000). No significant differences in growth were recorded on the different growth media (F = 0.801; p = 0.373).

### DNA sequence analysis

**Figure 3.**
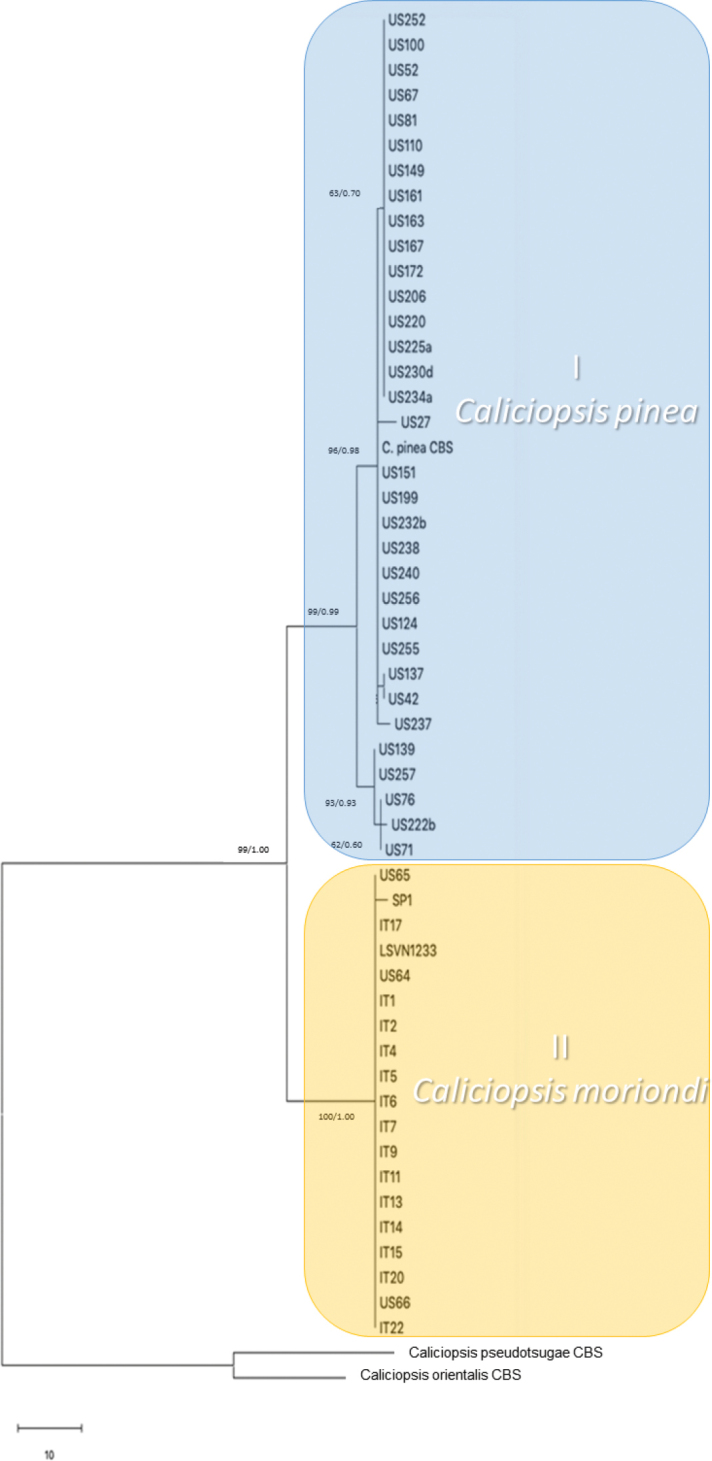
One of the most parsimonious trees (length = 137) from the combined sequence datasets of the ITS rDNA, Bt1 and EF1-α loci is shown (CI = 0.9444, RI = 0.99633, RC = 0.98178, HI = 0.94098). MP bootstraps and Bayesian posterior probabilities are indicated alongside the branches. *C.pseudotsugae* and *C.orientalis*EF1-α were selected as outgroup taxa.

**Figure 4.**
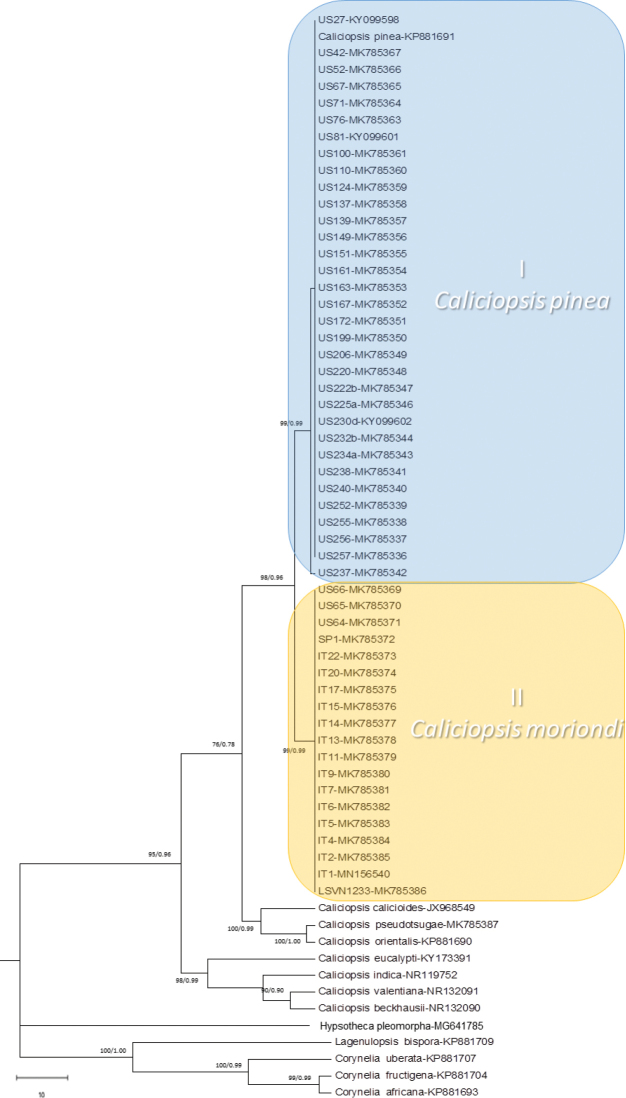
One of the most parsimonious trees of aligned ITS dataset (length = 168, CI = 0.721154, RI = 0.922043, RC = 0.762881, HI = 0.664935). The MP and Bayesian posterior probability are indicated next to the branches.

The final combined ITS–EF1-α –Bt1 data matrix of *Caliciopsis* included 53 ingroup and 2 outgroup sequences (length = 137, CI = 0.9444, RI = 0.99633, RC = 0.98178, HI = 0.940979) (Figure 3), comprising 1611 alignment characters, including gaps. Of these, 1434 characters were constant and 112 characters were parsimony informative (Figure 3). Single ITS, EF1-α and Bt1 datasets included, respectively, 458, 762 and 373 characters (Figure 4, Suppl. material 1: Figure S1 and Suppl. material 2: Figure S2)

Phylogenetic analysis, resulting in the most parsimonious tree from the concatenated dataset, showed that isolates, previously identified as *Caliciopsispinea*, based on morphology, grouped in two different clades. One of these clades (Clade I) included most of the US strains and the *C.pinea* isolate CBS 139.64. The other clade (Clade II) included all EU isolates and three US strains from *P.resinosa* from a single location in New Hampshire (US64, US65, US66). Maximum Parsimony and Bayesian Inference produced nearly identical topologies for all single locus datasets: ITS, which included different species of *Caliciopsis* and other Coryneliaceae (*Coryneliaafricana*, *C.fructigena*, *C.uberata* and *Lagenulopsisbispora*) (length = 168, CI = 0.721154, RI = 0.922043, RC = 0.762881, HI = 0.664935); Bt1 (length = 62, CI = 0.926829, RI = 0.98404, RC = 0.936428, HI = 0.912039); EF1-α gene (length = 66, CI = 0.9999, RI = 0.9998, RC = 0.9988, HI = 0.9888) (Figure 4, Suppl. material 1: Figure S1 and Suppl. material 2: Figure S2).

**Figure 5.**
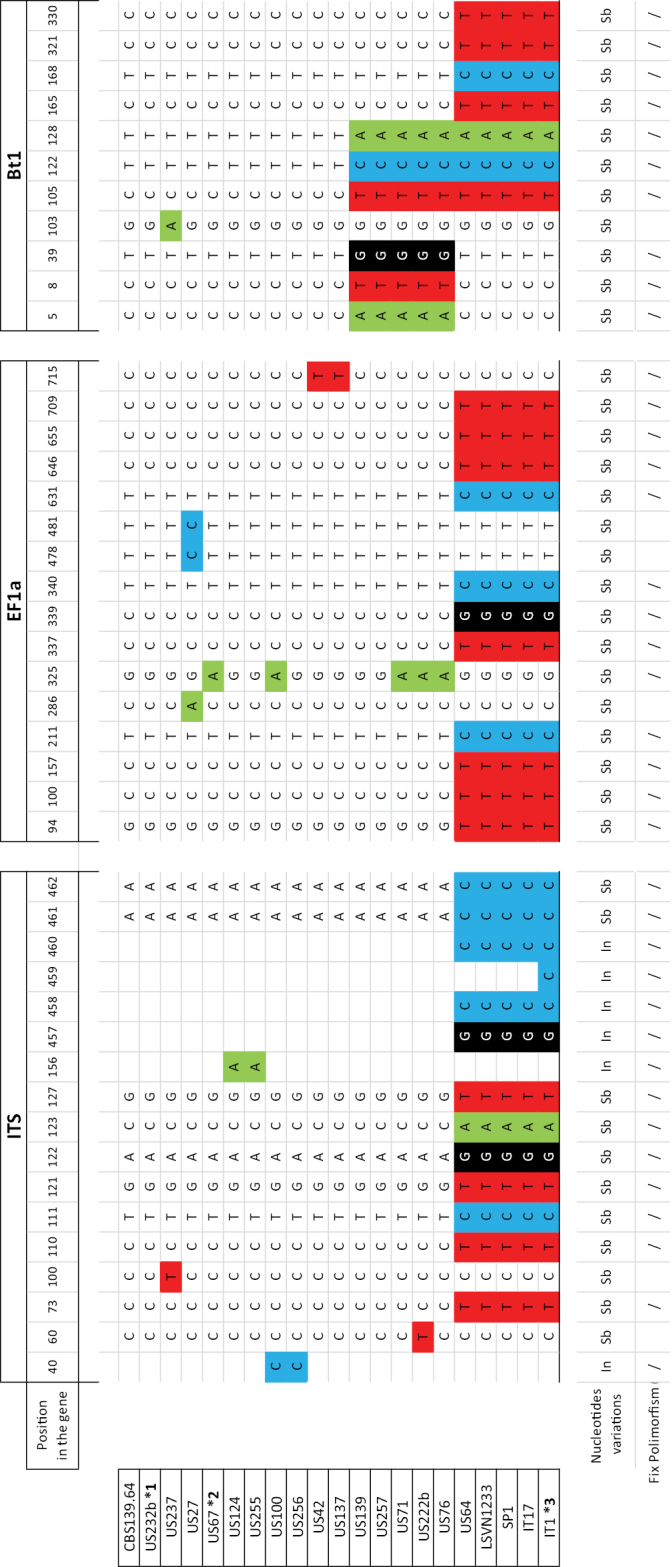
Polymorphic nucleotides from aligned sequence data of ITS, EF1-α and Bt1 loci showing the variation between isolates of *Caliciopsispinea* from US and isolates of *C.moriondi* from the US and EU. Different colours demark variation in bases found in the sequences. Variation type is reported in the bottom part: “Sb” is the abbreviation for base’s substitution; “In” is the abbreviation for base’s insertion. Fix polymorphisms are signalled with “/”. Sequences US232b, US67 and IT1 are respectively marked with *1, *2, *3 as representative for the following groups of sequences not reported in the figure: *1 (US151, US199, US238, US240, US232b); *2 (US52, US100, US81, US110, US149, US161, US163, US167, US172, US206, US220, US225a, US230d, US234a, US252); *3 (IT2, IT4, IT5, IT6, IT7, IT9, IT11, IT13, IT14, IT15, IT20, IT22, US66, US65)

Across the three loci sequenced, there were 31 ﬁxed polymorphisms separating Clade I from Clade II. Of these, 12 were in the ITS region, 11 in EF1-α and 7 in Bt1 (Figure 5). The USA isolates US64, US65 and US66 shared the same fixed polymorphisms present in Clade II samples. However, samples US64 together with isolates SP1, LSVN1233 and IT17 did not have the insertion in position 459 of ITS, which was one of the fixed polymorphisms in Clade II samples. Ten fixed polymorphisms were specific to Clade I. Of these, two were in the ITS, two in the EF1-α and six in the Bt1, where three in position 105, 122 and 128 were in common with isolates in Clade II. Fixed, unique polymorphisms were identified in all three loci, which produced congruent trees from the individual loci that separated Clade I (*C.pinea*) from Clade II isolates, suggesting that the latter isolates represent a novel species different from *C.pinea*.

## Taxonomy

### Caliciopsis moriondi

Taxon classificationFungiCorynelialesCoryneliaceae

N. Luchi, D. Migliorini & A. Santini sp. nov.

B29BE3DE-DDF5-5003-AE77-C22CC7FFC834

833212

Figures 1 , 2

#### Types.

Italy, Florence, Lastra a Signa, Carcheri, 43°71.58'N, 11°07.36'E, 110 m a.s.l., isolated from branches of *Pinusradiata*, 10 Oct. 2014, *leg.* N. Luchi, D. Migliorini & A. Santini, CBS 146717 (**holotype**); (IT1). ex-holotype sequences MN156540 (ITS), MK913586 (EF1-α), MN150120 (Bt1); duplicate deposited at Fungal Collection of the Institute for Sustainable Plant Protection-National Research Council (IT1; **isotype**). ITALY, Florence, Fucecchio, 43°47'17"N, 10°46'37"E, isolated from diseased *Pinusradiata*, 5 Dec. 2014, *leg.* N. Luchi, deposited at Fungal Collection of the Institute for Sustainable Plant Protection-National Research Council (IT22, **paratype**). ITALY, Florence, Lastra a Signa, Carcheri, 43°71.58'N, 11°07.36'E, isolated from diseased *Pinusradiata*, 10 Oct. 2014, *leg.* N. Luchi, deposited at Fungal Collection of the Institute for Sustainable Plant Protection-National Research Council (IT4, **paratype**). ITALY, Florence, Antella 43°44.00'N, 11°19.52'E, isolated from diseased *Pinusnigra*, 24 Nov. 2014, *leg.* D. Migliorini, deposited at Fungal Collection of the Institute for Sustainable Plant Protection-National Research Council (IT17, **paratype**). SPAIN, San Sebastián de Garabandal, 43°12.04'N, 4°25.25'W, isolated from diseased *Pinusradiata*, 25 May 2011, *leg.* P. Capretti, deposited at Fungal Collection of the Institute for Sustainable Plant Protection-National Research Council (SP1, **paratype**).

#### Description.

Stromata developing beneath the surface of host periderm as small, more or less circular structures, giving little external evidence of their presence at early stages. Continued growth causing the bark to break and the minute cushion-shaped stromata, developing a lobed appearance and increasing in diameter and thickness, in black short-stalked columnar ascomata. Ascomata mostly frequent protruding at the margin of cankers, single or double, rarely triple, stalks not branched, (0.45) 0.84 ± 0.02 (1.2) mm high and (51) 79 ± 2 (135) µm width. Ascigerous swelling, terminal, (106) 281 ± 8 (406) µm high and (81) 142 ± 5 (268) µm diameter, forming a brownish pulverulent mass of extruded ascospores. Asci bitunicate, clavate, 8-spored, slightly curved, pedicellate, (26) 37 ± 6 (53) µm long; pedicel 1–3 µm diameter; sporiferous part (12) 13 ± 0.4 (14.2) µm long and (5.3) 6.3 ± 0.4 (7.4) µm wide. Ascospores yellow-green colour, sub-globose to ellipsoidal and often aggregated in small masses, (3) 4.4 ± 0.07 (6.2) µm long and (1.8) 2.5 ± 0.04 (3.5) µm wide, brown when mature. Spermogonia sub-globose, papillate, sessile, aggregated below ascomatal tubes. Conidia unicellular, hyaline, smooth, slightly fusiform.

#### Culture characteristics.

Cultures incubated on 2% PDA, showed optimal temperature for growth at 20 °C, with slow-growth rate (1.4 mm/day). Colonies white appressed to the agar, circular to irregular, becoming fawn-colored with age, areas towards margin floccose; mycelium velutinous with funicolose areas or strongly funicolose in the inner and older parts of the mycelium. Reverse colony brownish, with brown diffusion zone in old cultures; branching septate hyphae, with frequent anastomoses and tips with dendroid branching.

#### Inoculation tests.

All isolates from Italy and residing in Clade II inoculated on seedlings gave rise to symptoms and lesions of variable length after six months. These were all significantly different to those of the controls (F = 119.21, p < 0.000; F = 60.84, p < 0.000, respectively). Inoculated plants did not show a crown dieback, but all had profuse resin production at the inoculation points. *Caliciopsismoriondi* fruiting bodies were clearly visible on *P.halepensis*, while no fructifications were seen in any of the other inoculated *Pinus* species.

The lengths of lesions caused by the inoculated isolates were significantly longer on *P.halepensis* (28.6 ± 9.04 mm) and *P.pinaster* (30.1 ± 7.13 mm) than on *P.pinea* (16.4 ± 3.16 mm) (F = 297.43, p < 0.000). *Caliciopsismoriondi* was successfully re-isolated from all the seedlings inoculated with the pathogen, while no *Caliciopsis* species were re-isolated from mock-inoculated seedlings.

#### Hosts and distribution.

Pathogen of pine trees *P.nigra*, *P.radiata* and *P.resinosa*, causing cankers and resin production in Europe (France, Italy, Spain) and North America (New Hampshire, USA).

#### Etymology.

The name *moriondi* honours Prof. Francesco Moriondo (1926–2014). Francesco Moriondo was the founder of forest pathology as a discipline distinct from plant pathology in Italy. In this respect, he preferred a more ecological view of the topic as opposed to the typical mechanistic approach. During his career, he encouraged many young students to consider the reasons for the appearance of symptoms on trees, rather than considering only the causative agents. He also emphasised the importance of minor pathogens in the ecosystem, of which *Caliciopsismoriondi* (then *C.pinea*) was one.

#### Notes.

*Caliciopsismoriondi* is commonly associated with a canker disease on *Pinus* spp. It differs subtly from *C.pinea*, based on morphological traits, including shorter ascomata, protruding and isolated from the stroma, rarely in groups of two-three, but never in more numerous groups, such as is common for *C.pinea* (Table 2).

## Discussion

This study included a large number of isolates previously believed to be *Caliciopsispinea*. Analysis of DNA sequences of the ITS, Bt1 and EF1-α regions clearly showed that these isolates represented two distinct taxa. One of these represented *C.pinea* and the other an undescribed species, which we have formally described here as *C.moriondi*.

*Caliciopsismoriondi* can be distinguished from *C.pinea* based on various morphological features including the length of the ascomata, as well as by their distribution on the stromata. In the absence of sequence data, previous authors confused isolates obtained in Europe with *C.pinea*, which was originally described from North America by Fitzpatrick (1920). *Caliciopsismoriondi* as the fungus is now known, has been found in Italy, France and Spain, mainly on *P.radiata* trees and, on one occasion, on *P.nigra*. Based on the wide sampling in this study, it appears likely that *C.pinea* does not occur in Europe.

Delatour (1969) described *Caliciopsispinea* as a weak pathogen by basing his assessment on inoculations of *P.pinaster* in France. The results of the present study suggest that it is more likely that this author was working with *C.moriondi*. This view is supported by the illustrations of *C.pinea* by Lanier (1965) showing ascomata very similar to those of *C.moriondi*.

*Caliciopsismoriondi* was able to cause only mild symptoms when inoculated on Mediterranean *Pinus* spp. in pathogenicity trials. The symptoms were most noticeable on *P.halepensis* and less severe on *P.pinaster* and *P.pinea*, confirming the results of Delatour (1969). *Caliciopsismoriondi* was able to produce ascocarps when inoculated on *P.halepensis*, but not on *P.pinaster* and *P.pinea*.

Interestingly, the European isolates of *Caliciopsismoriondi* were mainly found on *Pinusradiata*. Our inoculation tests, as well as those of Delatour (1969), suggest that the non-native *P.radiata* is more susceptible than Mediterranean *Pinus* spp. Unfortunately, *P.radiata* and *P.nigra* plants were not available when this pathogenicity test was undertaken. A further inoculation experiment on these two *Pinus* spp., which are widely planted in Europe, will be necessary in order to assess their susceptibility to *C.moriondi*.

The results of this study suggest that *Caliciopsismoriondi* is native to Europe. This is supported by the fact that it caused only mild symptoms on artificially inoculated European *Pinus* spp. Yet on naturally infected non-native *P.radiata*, it gave rise to symptoms similar to those caused by the pitch canker pathogen *Fusariumcircinatum*, which is an important quarantine pathogen in Europe and also commonly found on *P.radiata* (Capretti et al. 2013). Future studies will be necessary to determine whether infections on these trees are caused by *F.circinatum* or *C.moriondi* and the duplex real-time PCR assay developed and validated by Luchi et al. (2018) should be useful in this regard.

*Caliciopsismoriondi* and *C.pinea* are two vicariant species and it appears that the European *C.moriondi* has been accidentally introduced in North America. We hypothesise that this might have occurred at the end of the 1800s when European nurseries produced large volumes of *Pinus* spp. for the establishment of North American plantations (Maloy 1997). *Caliciopsismoriondi* could easily have moved on infected, but asymptomatic, seedlings at that stage.

The results of this study suggest that *Caliciopsispinea* is not present in Europe. Its pathogenicity on European pines has never been assessed. Since the beginning of the present century, there has been a renewed interest in this species due to the damage it causes to the plantations of *P.strobus* in the north-eastern United States (Munck et al. 2015, 2016). An accidental introduction of this species into Europe could pose a threat to European pine plantations and natural forests. Consequently, it will be important to assess the susceptibility of European *Pinus* spp. to this pathogen and to prepare an *ad hoc* pest risk assessment for it.

## Supplementary Material

XML Treatment for Caliciopsis moriondi

## Supplementary materials

Supplementary material 1 Figure S1. One of the most parsimonious trees from EF1-α sequence datasets Data type Alignment of genomic sequences Explanation note One of the most parsimonious trees from EF1-α gene sequence datasets is shown (length = 66, CI = 0.9999, RI = 0.9998, RC = 0.9988, HI = 0.9888). The MP and Bayesian posterior probability are indicated next to the branches. *C.pseudotsugae* and *C.orientalis* are used as outgroup. https://binary.pensoft.net/file/456660This dataset is made available under the Open Database License (http://opendatacommons.org/licenses/odbl/1.0/). The Open Database License (ODbL) is a license agreement intended to allow users to freely share, modify, and use this Dataset while maintaining this same freedom for others, provided that the original source and author(s) are credited.Duccio Migliorini, Nicola Luchi, Alessia Lucia Pepori, Francesco Pecori, Chiara Aglietti, Fabio Maccioni, Isabel Munck, Stephen Wyka, Kirk Broders, Michael J. Wingfield, Alberto Santini

Supplementary material 2 Figure S2. One of the most parsimonious trees from Bt1 sequence datasets Data type Alignment of genomic sequences Explanation note One of the most parsimonious trees from Bt1 sequence datasets is shown (CI = 0.9268, RI = 0.9840, RC = 0.936428, HI = 0.912039). The MP and Bayesian posterior probability are indicated next to the branches. *C.pseudotsugae* and *C.orientalis* are used as outgroup. https://binary.pensoft.net/file/456661This dataset is made available under the Open Database License (http://opendatacommons.org/licenses/odbl/1.0/). The Open Database License (ODbL) is a license agreement intended to allow users to freely share, modify, and use this Dataset while maintaining this same freedom for others, provided that the original source and author(s) are credited.Duccio Migliorini, Nicola Luchi, Alessia Lucia Pepori, Francesco Pecori, Chiara Aglietti, Fabio Maccioni, Isabel Munck, Stephen Wyka, Kirk Broders, Michael J. Wingfield, Alberto Santini
