# Supplementary figures and images for: Caliciopsismoriondi, a new species for a fungus long confused with the pine pathogen C.pinea
Source: MycoKeys. 2020 Sep 25;73:87–108. doi: 10.3897/mycokeys.73.53028 (PMC7532227; doi:10.3897/mycokeys.73.53028)

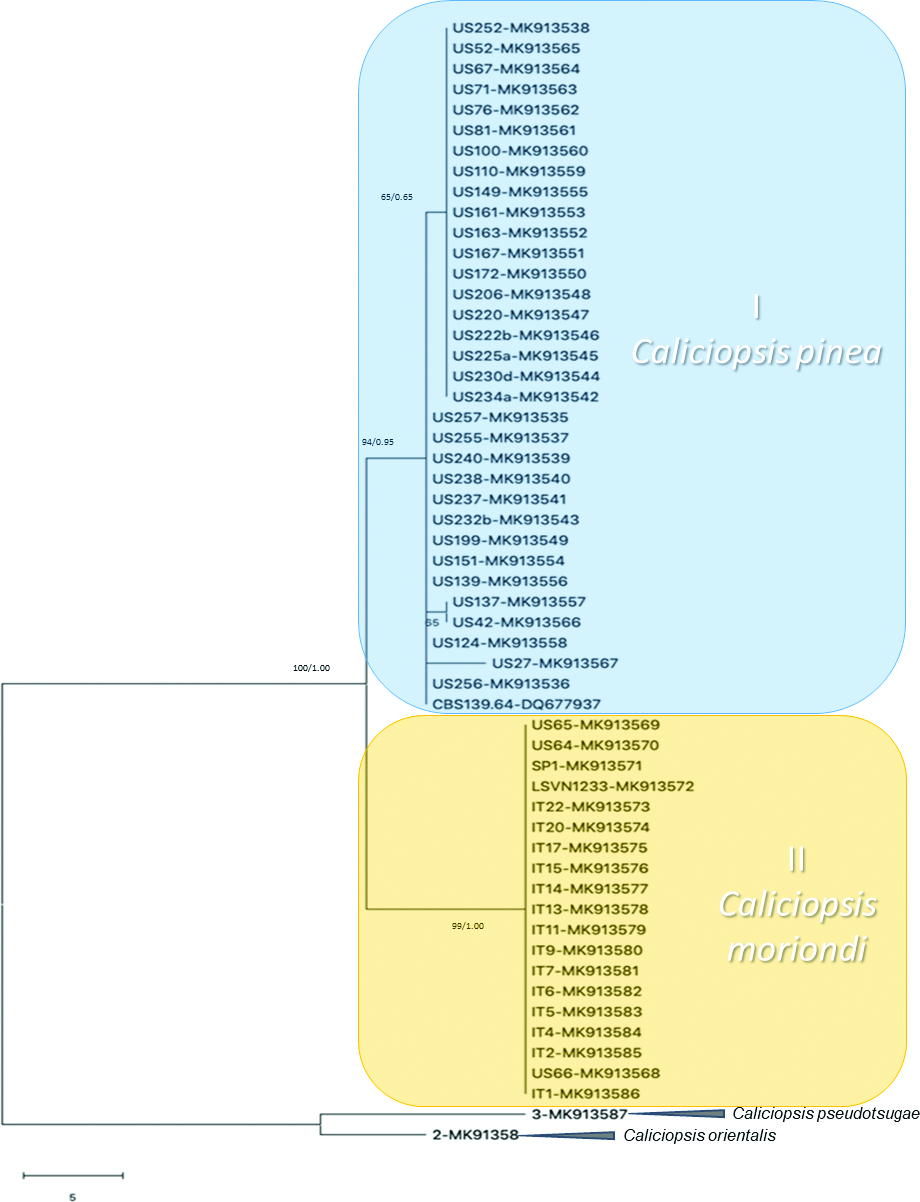

Supplement: Supplementary material 1 — Figure S1. One of the most parsimonious trees from EF1-α sequence datasets [file mycokeys-73-087-s001.tif]

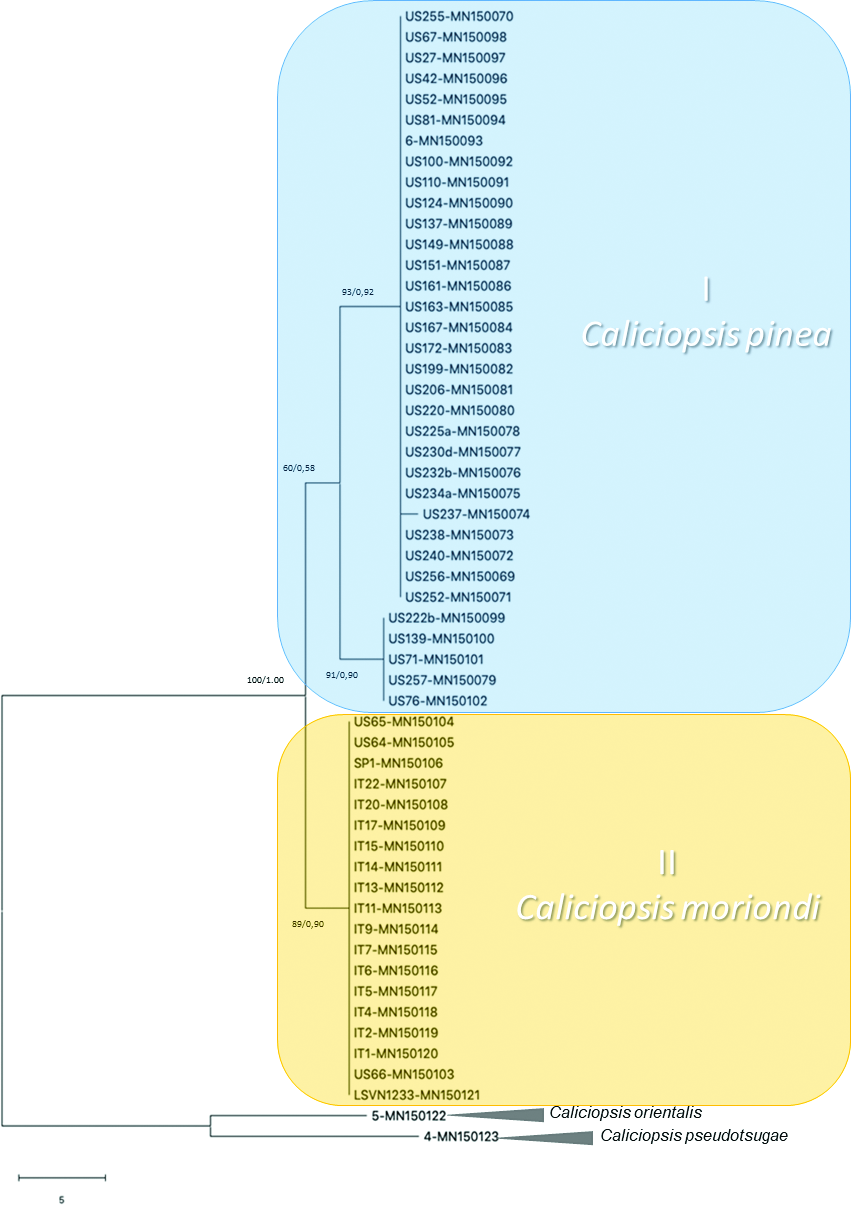

Supplement: Supplementary material 2 — Figure S2. One of the most parsimonious trees from Bt1 sequence datasets [file mycokeys-73-087-s002.tif]
